# The Effect of Pain Relief on Daily Physical Activity: In-Home Objective Physical Activity Assessment in Chronic Low Back Pain Patients after Paravertebral Spinal Block

**DOI:** 10.3390/s18093048

**Published:** 2018-09-12

**Authors:** Tzu Chuan Yen, Jane Mohler, Michael Dohm, Kaveh Laksari, Bijan Najafi, Nima Toosizadeh

**Affiliations:** 1 Arizona Center on Aging, Department of Medicine, University of Arizona, Tucson, AZ 85719, USA; tzuchuan-yen@uiowa.edu (T.C.Y.); jmohler@aging.arizona.edu (J.M.); 2 Carver College of Medicine, University of Iowa, Iowa City, IA 52242, USA; 3 Department of Biomedical Engineering, University of Arizona, Tucson, AZ 85719, USA; klaksari@email.arizona.edu; 4 Division of Geriatrics, General Internal Medicine and Palliative Medicine, Department of Medicine, University of Arizona, Tucson, AZ 85719, USA; 5 Department of Orthopaedic Surgery, University of Arizona, Tucson, AZ 85719, USA; mdboneman@aol.com; 6 Interdisciplinary Consortium on Advanced Motion Performance, Division of Vascular Surgery and Endovascular Therapy, Michael E DeBakey Department of Surgery, Baylor College of Medicine, Houston, TX 77030, USA; najafi.bijan@gmail.com

**Keywords:** gait, pain, back disorder, outcome evaluations, daily activity, fear of pain

## Abstract

This study evaluates the effect of paravertebral spinal injection (PSI), utilizing both subjective and objective assessments in chronic low back pain (LBP) associated with facet joint arthrosis over a one-month duration. Subjective questionnaires included the visual analogue scale (VAS) for pain, the Oswestry Disability Index, the Health Survey SF-12, and the short Falls Efficacy Scale International (FES-I). Objective assessments included in-clinic gait and Timed Up and Go (TUG) tests using wearable sensors, as well as 48 h daily physical activity (DPA) monitored using a chest-worn triaxial accelerometer. Subjective and objective measures were performed prior to treatment, immediately after the treatment, and one month after the treatment. Eight LBP patients were recruited for this study (mean age = 54 ± 13 years, body mass index = 31.41 ± 6.52 kg/m^2^, 50% males). Results show significant decrease in pain (~55%, *p* < 0.05) and disability (Oswestry scores, ~21%, *p* < 0.05). In-clinic gait and TUG were also significantly improved (~16% and ~18% faster walking and shorter TUG, *p* < 0.05); however, DPA, including the percentage of physical activities (walking and standing) and the number of steps, showed no significant change after PSI (*p >* 0.25; effect size ≤ 0.44). We hypothesize that DPA may continue to be truncated to an extent by conditioned fear-avoidance, a psychological state that may prevent increase in daily physical activity to avoid pain.

## 1. Introduction

Low back pain (LBP) is the second most common source of disability in the United States, affecting more than 80% of the population during their lifetime [1,2]. This can lead to costs reaching $200 billion annually [3]. Depending on the type of LBP, several types of treatment exist for reducing pain, including medication, exercise therapy, massage therapy, spinal manipulation, temperature treatment, transcutaneous electrical nerve stimulation, acupuncture, and spinal steroid injection [4,5]. Regardless of the type of intervention, evidence is needed to show the effect of any treatment in the clinic and at home, and whether immediate improvement in post-treatment physical function leads to improvement in daily physical activity (DPA). In the current study, we investigated how the effect of spinal injection, a common minimally-invasive pain reduction intervention, can reduce LBP symptoms and pain caused by lumbar facet joint arthrosis, and how these effects may enhance DPA.

Among its many causes, lumbar facet joint pain accounts for up to 75% of the patients who present with LBP [6]. One specific cause of LBP is lumbar degenerative facet osteoarthropathy, which is characterized by cartilage degradation and sclerosis in the subchondral bone at the synovial joint, leading to impingement of nerve roots as the cause of pain [6]. Among the most common treatments, paravertebral spinal injection (PSI) is often utilized to manage chronic LBP [7]. Spinal injections are categorized as epidural steroid (interlaminar or caudal), transforaminal epidural (selective nerve block), and paravertebral facet blocks (intra-articular or extra-articular medial or intermediate branch block). We investigated the utilization of paravertebral facet blocks and included both extra-articular and intra-articular as a PSI in order to encompass the medial and intermediate branch.

Although PSI has been commonly implemented for LBP treatment, there are few clinical trials pertaining to the use of lumbar PSI, and among those available, no consensus regarding effectiveness has been reached [8]. One study investigated transforaminal or caudal fluroscopically guided epidural steroid injections for lumbar spinal stenosis, which found that 32% of the patients reported pain relief beyond two months post-injection, along with 53% who confirmed functional ability improvement at an average of 1.5 years post-injection [9]. In another study specifically focusing on pain measures, 9 out of 30 patients who underwent fluoroscopically guided transforaminal epidural steroid injections for lumbar disc herniation reported complete pain relief after 24 weeks from the beginning of the study, which allowed for one injection every two weeks with a maximum of three injections [10]. In another study, functional disability measured by the Roland-Morris Disability Questionnaire was insignificantly improved after six weeks of fluoroscopically guided transforaminal or interlaminar epidural steroid injections for lumbar spinal stenosis compared to baseline [11]. Interestingly, one study assessed pain, disability, and physical impairment one week post-injection for lumbar spinal stenosis, but found that improvements in pain and function compared to baseline measurements did not positively correlate with the objective assessment of total activity over a period of seven days; no significant changes in physical performance were discovered with treatment using fluoroscopically guided epidural steroid injections [12]. Within our recent investigation, we showed that fluoroscopy guided PSI for LBP improved motor performance between one and three months, but most notably at one month post-injection [13]. According to these previous studies, decrease in pain is often observed after PSI but for varying lengths of time [9,10]. Inconsistency also exists in the reported functional ability and physical performance status following PSI [9,11,12,13]. Additionally, no study has compared changes in subjective and objective parameters, especially greater than or equal to a 30-month period after the treatment.

The primary aim of this study was to investigate the efficacy of paravertebral facet injection for degenerative facet osteoarthropathy, both subjectively and objectively over a period of one month, using wearable sensor technology to track DPA. Notable differences separating this study from previous investigations were the inclusion of previously unreported measures, the comparison of pain-related subjective and objective measures (both in-clinic and in-home measures), as well as the inclusion of patients with more diverse etiologies of LBP, which most accurately reflects the typical patient population. Specifically, we investigated: (1) how objectively measured DPA would change over the one-month period following spinal injection treatment; and (2) if and how much DPA measurement would be associated with post-treatment alterations in pain, disability, and objective in-clinic measures of gait and Timed Up and Go (TUG).

## 2. Materials and Methods

### 2.1. Participants

Participants with degenerative facet osteoarthropathy were recruited from the Southwestern tertiary integrated academic health care system based on clinical and radiologic diagnosis with plain film radiography, CT, and MRI images. The inclusion criteria were as follows: (1) LBP symptoms of at least three months prior to assessment; (2) 18 years of age or older; and (3) ability to walk 20 m without any assistive device. The exclusion criteria included the following: (1) prior spine, hip, or lower-extremity surgeries within one month of the spinal injection; (2) opioid use for pain; and/or (3) severe comorbidities that altered gait or balance performance, such as Parkinson’s disease, stroke, diabetic neuropathy, or peripheral vascular disease. We selected participants over a large age range, and also those with general facet joint pain, to represent a normal LBP patient population. The study was approved by the University of Arizona Institutional Review Board. Written informed consent according to the principles expressed in the Declaration of Helsinki [14] was obtained from all subjects before participation.

### 2.2. Paravertebral Facet Injection

Paravertebral facet injections were performed at the same facility by a single orthopaedic surgeon (MD) according to the North American Spine Society’s recommendations [15]. Under fluoroscopic guidance, participants were treated with 1 cc of Isovue-300, 3.5 cc of 1% plain lidocaine, 3.5 cc of 0.25% plain Marcaine, and 2 cc of 40 mg per cc of triamcinolone. The surgeon advanced the 10 cc syringe injectate while patients were prone on a radiolucent table. The spinal needle was placed at the center of the pedicle cephalad border for a medial branch block and an intermediate branch block, pericapsular, and intracapsular. Chloraprep was utilized both pre- and post-injection and adhesive bandages were placed on the points of entry afterwards. Two cc of injectate were deployed at each site. Patients ambulated immediately following the injection, and data collection began within two hours post-injection

### 2.3. Patient-Reported Outcome Measures (PROMs)

Participants were asked to report pain, health survey, and disability information in, at most, three sessions: pre-injection within three days prior to the procedure (baseline), immediately post-injection, and one month post-injection. The 10-point visual analog scale (VAS) [16] was used to assess pain at the exact moment in time, as well as average pain level across a prior two-week period. The Oswestry Disability Index for Functional Outcomes [17], the short Falls Efficacy Scale International (FES-I) [18], and the SF-12 Health Survey for General Health Status [19] were collected. Not all PROMs were collected at each time; the Oswestry Disability Index, two-week pain, FES-I, and health survey were only collected before the injection during the first visit, but were not repeated immediately after the treatment, because the questions referred to a prior two-week period.

### 2.4. Objective Motor Performance Measurements

**Figure 1:**
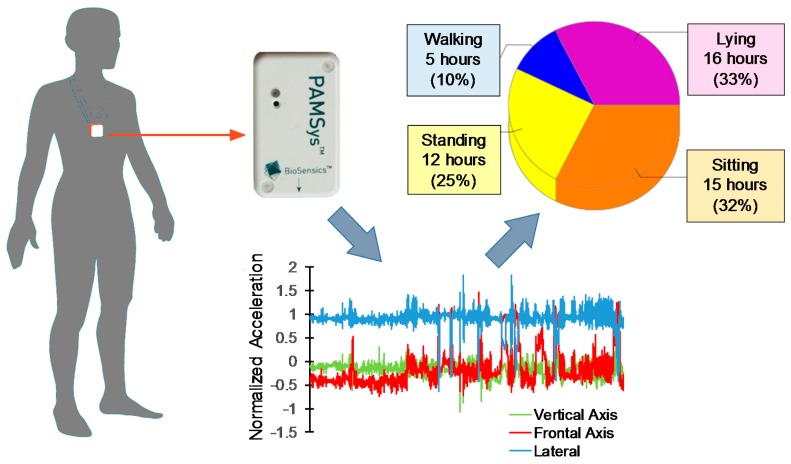
Triaxial accelerometer wearable sensor (PAMSys) was used to estimate the amount of physical activity and the number of steps. Results are presented for 48 h daily physical activity from one participant for baseline (pre-injection).

To measure DPA, a previously validated method involving a wearable triaxial accelerometer (sampling frequency = 50 Hz; PAMSys, BioSensics, Watertown, MA, USA) [20,21,22,23,24,25] was implemented. The wearable sensor was attached to a snuggly fit shirt in a mid-sternal pocket (see Figure 1). This system recorded accelerometer data, which were uploaded to the home-made software to estimate the duration and number of various spontaneous activities at home. Participants wore the shirt for three periods, including: (1) pre-injection (within three days before admission); (2) post-injection (within two hours post-injection); and (3) one month post-injection (within an hour after the one month post-injection visit). In each round, participants were instructed to wear the shirt for 48 h (two consecutive weekdays from Monday through Friday) and to remove it only when showering. Our previous studies suggested that a 48 h duration was an optimum tradeoff between reliability and adherence to continuously wearing the sensor [23,26]. Previously validated DPA variables derived from PAMSys data included the following: amount of time sitting, standing, walking, and lying down within 48 h; percentage of each posture (sitting, standing, walking, and lying); maximum walking duration and walking duration variability (i.e., variability in duration of walking bouts) within 48 h; duration of sit-to-stand and stand-to-sit transitions; and gait parameters including steps, cadence, and the number of walking episodes.

In-clinic motor performance, including normal gait and the TUG test, was performed within 20–30 min following the spinal injection using a validated system (sampling frequency = 100 Hz, LEGSys, BioSensics, Watertown, MA, USA). Briefly, three-dimensional acceleration and angular velocity of shanks, thighs, and the trunk were measured using five wearable sensors, each including a triaxial accelerometer and a triaxial gyroscope to derive gait and TUG outcome measures following procedures identical to those explained in earlier studies (see Table 1 and Table 2). Sensors were attached to the shank above the ankle, to the thigh above the knee, and to the lower back in the lumbar region. Parameters related to gait included: (1) gait speed: the distance travelled divided by the total walking time; (2) stride length: the mean value of the distance traveled by the same limb in one gait cycle; (3) gait cycle time: the time between two successive heel contacts by the same foot; and (4) speed variability: the standard deviation divided by the mean gait speed across all gait cycles [27,28]. In addition, for the TUG test, the total time required to complete the test was recorded [29].

### 2.5. Data Analysis

Data were analyzed in JMP (Version 14, SAS Institute Inc., Cary, NC, USA), which is a commercialized computer program for statistical analysis. Statistical significance was concluded when *p* ≤ 0.05. For continuous data over the one-month period (pain, DPA parameters from PAMSys, gait, and TUG), the repeated measure analysis of variance (ANOVA) tests, adjusted for age, gender, and body mass index (BMI), was utilized. The Student’s *t*-test was used to compare pre-injection versus immediate post-injection parameters (pain and DPA parameters from PAMSys), as well as pre-injection versus one month post-injection parameters (pain, SF-12, FES-I, and Oswestry). Means with standard deviation and 95% confidence intervals for data, as well as the Cohen’s effect size for Student’s *t*-test and the Eta-squared for ANOVA, were calculated.

## 3. Results

### 3.1. Participants

Eight LBP patients (four males and four females) were recruited for this study, with two lost for the one-month follow-up. Mean (SD: standard deviation) age, BMI, height, and weight of the participants were 54 (13) years, 31.41 (6.52) kg/m^2^, 168.87 (10.06) cm, and 90.32 (22.50) kg, respectively.

### 3.2. Changes in PROMs Following Spinal Injection

Pain at the moment and Oswestry scores were significantly improved from baseline to the one-month follow-up; pain and Oswestry scores decreased (improved) by 46% and 21%, respectively (*p* < 0.05, Table 1). Pain at the moment decreased by 62% at immediate follow-up, and average pain in the last two weeks was also significantly decreased by 48% at the one-month follow-up. Although not significant, comparing one-month and baseline results, the SF-12 health survey and short FES-I fall risk assessments showed trends of improvements (*p >* 0.07).

### 3.3. Changes in DPA (PAMSys) Data

No significant difference was found in any of the DPA parameters immediately or after one month following spinal injection compared to baseline measurements (*p >* 0.25, Table 2).

### 3.4. Changes in Motor Performance Following Spinal Injection

Significant changes were observed from baseline to immediate and one-month follow-up for gait speed, gait cycle time, and total TUG duration (*p* < 0.05, Table 3). Gait speed increased by ~24% percent, and both gait cycle time and total TUG duration decreased by ~9% and ~21%, respectively, after one month post-injection compared to the baseline. Of note, one-month follow-up gait data were missed for two patient.

## 4. Discussion

### 4.1. Differences between Subjective and Objective In-Clinic and In-Home LBP Improvements

We investigated how removal of LBP using PSI resulted in decreased disability and improved mobility in performing daily activities. Our results suggested that although participants claimed reduced pain and showed improvements in in-clinic function measures (gait and TUG) post-injection, similar trends of improvements were not observed in DPA measures.

Pain in general decreases patients’ quality of life depending on its extent, duration, and intensity [30]. With regard to LBP, pain and disability are often measured using subjective self-assessments and their relationships with a patient’s quality of life is significant albeit weak due to other psychosocial factors [31]. LBP manifests in altered movement patterns when walking or changing positions, and may interfere with both activities of daily living and work-related functions [31,32]. Along with in-clinic measures of function that have been extensively studied for LBP treatments [33,34], measures of daily activities can be utilized to objectively evaluate disability due to pain. We previously showed that fluoroscopy guided PSI for LBP improved in-clinic motor performance, which could last between one to three months [13].

Considering subjective evaluations of PROMs, pain and disability, as measured by the visual analog scale and the Oswestry Disability Index, were significantly improved in patients after PSI (see Table 1). This result was consistent with previous work investigating transforaminal or caudal fluoroscopically guided epidural steroid injection for lumbar spinal stenosis, which reported 32% pain relief beyond two months post-injection, along with 53% of patients who confirmed functional ability improvement within an average period of 1.5 years post-injection [9].

Although several studies have reported subjective pain reduction after spinal injection, few investigated both subjective and objective measures. One study assessed pain, disability, and physical impairment one week post-injection for lumbar spinal stenosis. Although a different type of spinal disorder was targeted in that study, interestingly, and similar to our findings, they found that improvements in pain and function compared to baseline measurement did not positively correlate with the objective assessment of total DPA over a period of seven days [12]; no significant changes in physical performance including daily steps, maximum minutes of continuous activity, and percentage of total counts in the light, moderate, and high range of intensity were discovered with treatment using fluoroscopically guided PSI [12]. In contrast to our study, however, they did not measure in-clinic gait parameters nor did they measure parameters at one month post-injection.

According to current results, while immediate in-clinic assessments of gait and TUG revealed significant differences regarding gait speed, gait cycle time, and total TUG duration, no significant change in DPA for a duration of two days was observed at baseline, at the immediate follow-up, or at the one-month follow-up after the treatment (see Table 2 and Table 3). This disparity between in-clinic and at-home measures may be explained by the fear-avoidance phenomenon, in which patients defer from performing movement or activity because of change in muscle activity, deconditioning, or guarded movement as a result of chronic pain [35]. These immediate changes occur to avoid pain, and can become long-lasting and difficult to modify, as fewer opportunities might exist to change this acquired adaptation to prevent physical harm when it is chronic [35]. Interestingly, previous work showed that fear-avoidance is more strongly correlated with disability and work loss in the past year when compared with pain measures such as pain severity and duration [36]. Although we did not directly measure fear-avoidance in this study, our results suggest that while in-clinic motor performance (measured by gait and TUG parameters) and the level of pain do improve, individuals may be conditioned to maintain a modified level of physical performance due to persistent fear-avoidance.

### 4.2. Limitations and Future Directions

The study is limited by its sample size as well as the duration of follow-up. In future studies, more patients of disparate ages with diverse origins of LBP should be included to increase generalizability of the current findings. Specifically, follow-up should extend to a minimum duration of one year post-injection to better understand the time lag between pain relief and improvements in DPA. Several factors may positively or negatively skew the amount of DPA, such as weather conditions, special daily events, or daily personal mood. Therefore, in addition to quantitative measures of DPA, future studies with more reliable parameters related to quality of daily activities should be investigated, such as walking symmetry, variability, and regularity. Furthermore, a control group with patients who have no LBP could be used to determine if the severity of the chronic LBP correlates with the amount of time it takes to improve DPA. Additionally, in future studies, changes in DPA due to other types of neuromuscular disorders should be investigated.

### 4.3. Clinical Implications

Subjective measures of pain and disability or even in-clinic measures of motor function (gait and TUG) after PSI for chronic LBP may not correlate with short-term (one-month) improvements in DPA. Clinically, chronic LBP patients may not expect an immediate increase in DPA immediately post-injection. Hypothetically, differences between in-home activity and in-clinic measures of motor function may be related to fear of pain (psychological pain) and could affect patients’ ability to perform activities of daily living. Future studies should include measures of fear-avoidance to better understand the underlying mechanism of DPA improvements. In addition, consideration of adjunctive cognitive behavioral therapy with therapists or coaches could help address thoughts, emotions, and behaviors related to LBP by examining unhelpful thinking patterns and devising new ways of reaching optimal DPA goals for patients undergoing PSI for LBP.

## Figures and Tables

**Table 1:** Changes in patient-reported outcome measures (PROMs) over time. Results from repeated measure ANOVA (or *t*-test for baseline versus one-month follow-up comparisons) are presented. Mean (SD: standard deviation), *p*-values, confidence intervals (CIs), and effect sizes are presented. An asterisk indicates statistical significance.

	Baseline	Immediate FU	One-Month FU	*p*-Value	CI (Upper)	CI (Lower)	Effect Size
**Pain at the moment, 0–10 (SD)**	5.63 (3.42)	2.13 (2.01)	2.92 (2.65)	<0.05 *	−0.42	−4.74	0.81
**Pain in two weeks, 0–10 (SD)**	6.75 (2.76)	-	3.50 (2.72)	0.05 *	0.07	−5.73	1.01
**Oswestry, percentage (SD)**	41.8 (15.5)	-	33.0 (17.6)	<0.05 *	−0.029	−0.13	0.47
**SF-12, PCS (SD)**	25.2 (4.7)	-	32.2 (13.0)	0.08	15.03	−1.30	0.65
**SF-12, MCS (SD)**	40.2 (17.1)	-	49.8 (6.4)	0.07	17.31	−0.85	0.89
**Short FES-I, 7–28 (SD)**	18.3 (6.1)	-	15.2 (3.9)	0.29	2.96	−7.96	0.43

FU: follow-up; PCS: Physical Health Composite Scale; MCS: Mental Health Composite Scale; FES-I: Falls Efficacy Scale-International.

**Table 2:** Mean (SD: standard deviation) of objective 48 h daily physical activity measurements. *p*-value for Student’s *t*-test of baseline and immediate follow-up comparison, *p*-value for repeated measure ANOVA, and effect size for ANOVA are reported. An asterisk indicates statistical significance.

*Postural Summary*	Baseline	Immediate FU	One-Month FU	*p*-Value (*t*-Test)	*p*-Value (ANOVA)	Effect Size (ANOVA)
**Sitting, min (SD)**	866.09 (421.10)	974.66 (606.42)	970.98 (462.76)	0.46	0.54	0.10
**Standing, min (SD)**	465.43 (96.66)	404.60 (156.06)	595.07 (269.72)	0.33	0.25	0.44
**Walking, min (SD)**	136.58 (66.35)	141.44 (49.48)	122.91 (92.87)	0.90	0.79	0.11
**Maximum Walking Duration (s)**	290.38 (105.33)	353.75 (222.35)	251.67 (55.90)	0.50	0.74	0.33
**Lying, min (SD)**	1411.27 (386.41)	1348.46 (696.76)	1190.72 (382.21)	0.73	0.37	0.18
**% Sitting (SD)**	30.08 (14.62)	33.94 (21.02)	33.72 (16.07)	0.44	0.54	0.10
**% Standing (SD)**	15.16 (3.36)	14.14 (5.60)	20.66 (9.36)	0.35	0.25	0.43
**% Walking (SD)**	4.74 (2.31)	4.94 (1.76)	4.27 (3.22)	0.88	0.79	0.11
**% Lying (SD)**	49.01 (13.41)	46.99 (24.17)	41.35 (13.28)	0.74	0.37	0.18
*Walking Characterizing*						
**Total Steps (SD)**	7060.50 (3650.414)	7068.38 (2902.22)	5429.83 (4116.34)	0.76	0.43	0.20
**Walking Duration Variability (%)**	106.99 (12.78)	114.77 (30.73)	103.17 (10.71)	0.58	0.80	0.26
**Episode Cadence Average, steps/min (SD)**	67.16 (2.96)	67.08 (7.42)	66.45 (4.17)	0.98	0.87	0.06

FU: follow-up; ANOVA: analysis of variance.

**Table 3:** Results from repeated measure ANOVA for gait and TUG parameters. Mean (SD: standard deviation), *p*-values, confidence intervals (CIs), and effect sizes are presented. An asterisk indicates statistical significance.

	Baseline	Immediate FU	One-Month FU	*p*-Value	CI (Upper)	CI (Lower)	Effect Size
*Normal Gait*							
**Gait speed, m/s (SD)**	0.96 (0.24)	1.02 (0.22)	1.19 (0.14)	<0.05 *	0.19	0.03	0.44
**Stride length, m (SD)**	1.18 (0.19)	1.20 (0.20)	1.35 (0.14)	0.06	0.15	−0.002	0.39
**Gait cycle time, s (SD)**	1.27 (0.11)	1.21 (0.07)	1.15 (0.09)	<0.01 *	−0.02	−0.11	0.49
**Speed variability, % (SD)**	3.78 (3.17)	2.59 (1.47)	1.18 (0.57)	0.07	0.11	−2.60	0.58
**Cadence (steps/min)**	94.98 (4.27)	99.88 (3.00)	104.54 (4.29)	0.03 *	0.38	5.40	0.48
*TUG Test*							
**Total TUG duration, s (SD)**	14.25 (3.90)	12.17 (2.67)	11.27 (1.42)	<0.05 *	−20.34	−269.68	0.46

TUG: Timed Up and Go.
